# Validation of Cytomegalovirus Immune Competence Assays for the Characterization of CD8^+^ T Cell Responses Posttransplant

**DOI:** 10.1155/2012/451059

**Published:** 2012-12-16

**Authors:** Eugene V. Ravkov, Igor Y. Pavlov, Kimberly E. Hanson, Julio C. Delgado

**Affiliations:** Department of Pathology, ARUP Institute for Clinical and Experimental Pathology, School of Medicine, University of Utah, 500 Chipeta Way, Salt Lake City, UT 84108, USA

## Abstract

Cytomegalovirus (CMV) infection is one of the most important infectious complications of transplantation. Monitoring CMV-specific CD8 T cell immunity is useful for predicting active CMV infection and for directing targeted antiviral therapy. In this study, we examined four basic parameters for validation of CMV-specific tetramer staining and peptide stimulation assays that cover five most frequent HLA class I alleles. We also examined the potential use of CMV-specific CD8^+^ T cell numbers and functional and cytolytic responses in two autologous HSCT recipients treated for multiple myeloma. The coefficient of variation (CV %) of the precision within assays was 3.1−24% for HLA-tetramer staining, 2.5−47% for IFN-**γ**, and 3.4−59.7% for CD107a/b production upon peptide stimulation. The precision between assays was 5−26% for tetramer staining, 4−24% for IFN-**γ**, and 5−48% for CD107a/b. The limit of detection was 0.1−0.23 cells/**μ**L of blood for tetramer staining, 0−0.23 cell/**μ**L for IFN-**γ**, and 0.11−0.98 cells/**μ**L for CD107a/b. The assays were linear and specific. The reference interval with 95% confidence level was 0−18 cells/**μ**L for tetramer staining, 0−2 cells/**μ**L for IFN-**γ**, and 0–3 cells/**μ**L for CD107a/b. Our results provide acceptable measures of test performance for CMV immune competence assays for the characterization of CD8^+^ T cell responses posttransplant measured in the absolute cell count per **μ**L of blood.

## 1. Introduction

Clinical cytomegalovirus (CMV) is a significant cause of morbidity and mortality in patients who have undergone allogeneic hematopoietic stem cell transplantation (HSCT) or solid organ transplantation (SOT), despite the availability of antiviral treatments [1–3]. Like all herpes viruses, the primary CMV infection results in dissemination to almost every organ and establishment of asymptomatic viral latency in the immune-competent host [4]. CMV exposure is very common in the general population. Depending on demographic and geographic parameters, CMV seroprevalence rates range from 40 to 100% [5].

Cytotoxic CD8^+^ T cells play an important role in the control of acute CMV infection and in the maintenance of low viral loads during CMV latency [6, 7]. This control is mediated in an HLA class I restricted manner, in which CMV-specific CD8^+^ T cells specifically recognize an infected target via CD8^+^ T cell T-cell receptor (TCR) and HLA molecules presenting specific viral epitopes. After this specific recognition, CD8^+^ T cells become activated followed by massive proliferation and exertion of effector function toward infected cells. Inflammatory cytokines, including IFN-*γ*, in conjunction with a number of other molecules involved in degranulation of preformed lytic granules (e.g., CD107a and CD107b) are involved in the direct killing [8, 9].

Prophylactic antiviral treatments are effective at preventing invasive CMV disease posttransplant, but are typically administered for a limited time due to the cumulative drug toxicity and cost [10]. Consequently, after prophylaxis is stopped, there is a risk of CMV reactivation and/or disease development in immune-suppressed CMV seropositive patients [11]. Previous studies have shown that CMV disease develops in more than half of HSCT patients lacking detectable anti-CMV T-cell responses [12]. Accurate monitoring and quantification of CMV specific CD8^+^ T cell immunity posttransplant has the potential to revolutionize the way antiviral therapies are utilized during the posttransplant period. 

HLA class I tetramers are an important tool enabling physical enumeration and phenotypic characterization of antigen-specific CD8^+^ T cells [13, 14]. In addition, *in vitro* peptide stimulation followed by intracellular staining for IFN-*γ* and CD107a/b is a well-established method to measure the functional and cytotolytic characteristics of these cells correspondingly [9, 15]. The purpose of our study is to validate CMV-specific HLA class I tetramer staining and peptide stimulation-based functional and cytolytic assays that cover the most common HLA class I alleles in the general population. We have examined four basic parameters of assay validation (linearity, precision, sensitivity, and specificity) and presented reference ranges of CMV-specific CD8^+^ T cells and their numbers producing IFN-*γ* and CD107a/b molecules upon peptide stimulation. In addition, we present examples of the utility of the assays by evaluating CMV-specific CD8^+^ T cell immune status in patients undergoing HSCT. 

## 2. Methods

### 2.1. Donors and Samples

Whole blood specimens were obtained from healthy, CMV-seropositive adult donors (*n* = 33), who carried either HLA A*01:01, A*02:01, B*07:02, B*08:01, and/or B*35:01 alleles. The median age was 37 years old (range, 23–66); 11 females and 22 males. We also obtained samples from multiple myeloma patients undergoing tandem autologous HSCT. These subjects were also being screened weekly for CMV reactivation using an inhouse developed real-time quantitative CMV PCR assay. All blood draws were carried out according to the approved guideline procedures established by ARUP laboratories and the University of Utah Hospital. The blood samples were provided in heparin-anticoagulant tubes. Peripheral Blood Mononuclear Cells (PBMC) were isolated from blood samples over Ficoll-Paque Premium medium (GE Healthcare). All patient samples included in this study were identified according to protocol 7275, approved by the University of Utah Institutional Review Board, in order to meet the patient confidentiality guidelines of the Health Information Portability and Accountability Act.

### 2.2. Enumeration of CMV-Specific CD8^+^ T Cells with HLA-Tetramers and Count Beads

Enumeration of the CMV-specific CD8^+^ T cells was carried out on whole blood (50 *μ*L), using a panel of fluorescently labeled antibodies and either of HLA-tetramer reagents restricted to HLA A*01:01, A*02:01, B*07:02, B*08:01 and/or B*35:01 alleles presented in Table 1. CD3 FITC, CD8^+^ PE-Cy5 were purchased from BD Biosciences; the tetramers were obtained from Beckman-Coulter. The amount of the reagents was used according to the manufacturer. Following staining, the whole blood samples were treated with 500 *μ*L of BD lysing solution (BD Biosciences) to remove red blood cells and then transferred into tubes with counting beads (BD Trucount Tubes, BD Biosciences). The stained cells were analyzed on the FACS Canto II flow cytometer. Data were collected in four different dot plots with gates set to register events of three cell populations and beads. The forward scatter (FSC) versus side scatter (SSC) dot plot was used for setting up a gate on the lymphocyte population, which could be distinctly recognized by the size and granularity. The CD3 FITC versus CD8^+^ PE-Cy5 dot plot originated from the lymphocyte gate and was set to collect events corresponding to CD3^+^CD8^+^ T cell population. The CMV-specific HLA-tetramer versus CD8^+^ dot plot was derived from the CD3^+^CD8^+^ T cell gate to collect CMV-specific CD8^+^ T cells by placing a single gate above CD8^+^ and CMV HLA-tetramer positive cell population. The fourth dot plot, the side scatter versus CD8^+^, was included to collect the counting beads located in the upper right side of the plot.

### 2.3. CMV-Specific Functional and Cytotolytic Assay

 Assessments of T-cell function and cytoxicity were carried out in parallel with the HLA-tetramer staining of whole blood, using previously published protocols [15, 18]. PBMC were isolated from the same whole blood sample used for determining the absolute numbers of HLA-tetramer^+^ and CD3^+^CD8^+^ T cells. The antigen stimulation was achieved by adding a CMV-specific peptide (10 *μ*g/mL), cognate to its allele, and CD28 (1 *μ*g) and CD49d (1 *μ*g) costimulatory antibodies (eBioscience) to a culture with isolated PBMC (1 × 10^6^ cells). In order to facilitate the intracellular accumulation of IFN-*γ* and prevent recirculation of CD107a and CD107b during stimulation, the culture was supplemented with Brefeldin A (BFA) and monensin during the last 4 hours of activation. We also added CMV-specific HLA-tetramer to trace both responsive and unresponsive CMV-specific CD8^+^ T cells. After total of 6 hours stimulation, the cells were collected by centrifugation and stained with CD8^+^, CD107a, and CD107b fluorochrome-conjugated monoclonal antibodies. Detection of IFN-*γ* was achieved by washing and permeabilization with a saponin-based buffer (BD Cytofix/Cytoperm, BD Biosciences) and subsequent staining with IFN-*γ* APC-conjugated antibody. CD107a and CD107b FITC-conjugated antibodies were purchased from eBioscience; IFN-*γ* APC was bought from BD Biosciences. The stained cells were analyzed on the FACSCanto II flow cytometer. Data were acquired in four different dot plots with gates set to register events of 4 cell populations. The forward scatter (FSC) versus side scatter (SSC) dot plot was used for setting up a gate on the lymphocyte population. The SSC versus CD8^+^ PE-Cy5 dot plot derived from the lymphocyte gated population and was set to collect events representing CD8^+^ T cells. The CMV-specific HLA-tetramer versus IFN-*γ* APC dot plot was derived from the CD8^+^ T cell gate to collect CMV-specific CD8^+^ T cells producing IFN-*γ* by placing a four quadrant gate. The forth dot plot CMV-specific HLA-tetramer versus CD107a/b FITC dot plot was also derived from the CD8^+^ T cell gate to register CMV-specific CD8^+^ T cells expressing the cytolytic proteins.

### 2.4. Determining the Absolute Count of CMV-Specific CD8^+^ T Cells

The absolute count of CMV-specific CD8^+^ T cells per 1 *μ*L of blood was determined manually, according to the BD Truecount Tubes (BD Bioscience, cat. no. 340334) instructions, using the following equation: [no. of events in Tetramer^+^ gate]/[no. of events in beads gate] × [no. of beads per test tube]/[test volume of whole blood]. This assay also determined the absolute count of lymphocytes and CD3^+^CD8^+^ cells using the following formulas: [no. of events in Lymphocyte gate]/[no. of events in beads gate] × no. [of beads per test tube]/[test volume of whole blood], and [no. of events in CD3^+^CD8^+^ gate]/[no. of events in beads gate] × no. [of beads per test tube]/[test volume of whole blood]. The absolute count of CMV-specific CD8^+^ T cells producing either IFN-*γ* or CD107a/b relied on the data obtained from the enumeration of CMV-specific CD8^+^ T cells using counting beads combined with the cell frequencies obtained from the functional and cytolytic assay: IFN-*γ* ^+^Tet^+^ absolute count = [CD8^+^ T cells per *μ*L of blood] × [no. events for IFN-*γ* ^+^Tet^+^]/[no. event for CD8^+^ T cells], [CD107a/b^+^Tet^+^ absolute count] = [CD8^+^ T cells per *μ*L of blood] × [no. events for IFN-*γ* ^+^Tet^+^]/[no. event for CD8^+^ T cells].

### 2.5. Validation Parameters

Validation experiments were performed according to protocols recommended by the Clinical and Laboratory Standards Institute with minor modifications [http://www.clsi.org/]. Briefly, linearity was determined by serial dilution of PBMCs, isolated from CMV-seropositive, and HLA-matched donors, which were then either stained with tetramer or stimulated with peptide. The experiments were carried out in triplicates. Precision within assays was performed in five replicates. Precision between assays was determined from three independent experiments that were carried out in triplicates. Analytical sensitivity for both tetramer staining and functional and cytotoxicity assays was determined using either whole blood or isolated PBMC from three CMV-seronegative donors. The limit of detection was calculated as a concentration of average of positive cells between donors plus 2 standard deviations. Analytical specificity was evaluated using samples from HLA-mismatched and HLA-matched healthy donors. The reference ranges for both assays were established using samples from 33 healthy CMV-seropositive donors. 

### 2.6. Statistical Analyses

The data for linearity, precision within an assay and between assays were analyzed using EP Evaluator software (David G. Rhoads Associates, Inc.). The reference ranges data analysis was carried out with R software (The R Foundation for Statistical Computing), [http://www.R-project.org/], by bootstrapping 10,000 times with reference interval of 95%.

## 3. Results

### 3.1. Precision within Assays

In order to determine the extent of variability within the HLA-tetramer staining and CMV-peptide stimulation assays (intra-assay), we used whole blood samples drawn from 5 healthy CMV-seropositive donors with the HLA class I restriction representing HLA-A*01:01, HLA-A*02:01, HLA-B*07:02, HLA-B*08:01, and HLA-B*35:01 alleles. The collected data derives from the experiments carried out in 5 replicates on samples from each donor in both assays. We determined the average and a standard deviation (SD) using the absolute numbers of CMV-specific CD8^+^ T cells and those producing IFN-*γ* and CD107a/b, and then calculated the coefficient of variation (CV%).  Table 2 shows the results of the experiments. The variability within HLA-tetramer staining assay was in range between 3.1 and 24%. Similar pattern was observed in CMV-peptide stimulated samples. CMV-specific CD8^+^ T producing IFN-*γ* cells showed CV% range from 2.5 to 47%, while the numbers for CD107a/b positive cells were between 3.4 and 59.7%.

### 3.2. Precision between Assays

The purpose of determining the precision between assays (interassay) is to ascertain the variability of a given assay performed at different time points. We used the same donors and the overall experiment outline, except that the assays themselves were carried out separately three times in either 3 or 5 replicates. The average numbers of cells/*μ*L of whole blood were determined for each of the 3 runs of HLA-tetramer staining and CMV-peptide stimulation assays. We then used these numbers to calculate the average and SD values of all 3 runs and used them to determine CV% of interassays. The results are shown in Table 3. CV% of CMV-specific CD8^+^ T cells determined by tetramer staining of whole blood was in range from 4 to 12%. The CV% for IFN-*γ* and CD107a/b producing cells were from 3 to 15% and from 2 to 48% correspondingly. 

### 3.3. Linearity

**Figure 1:**
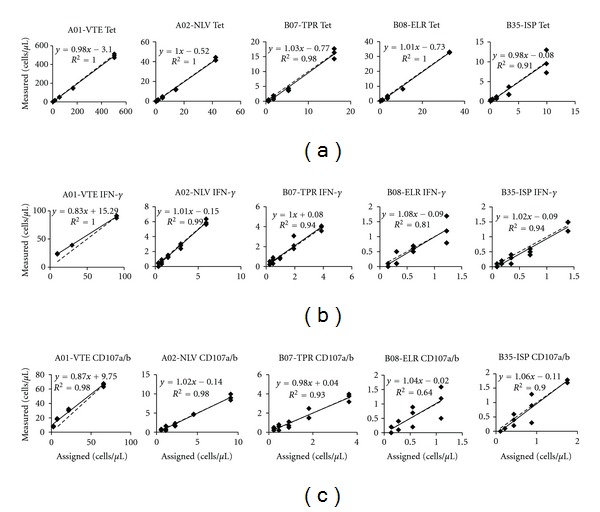
Linearity of tetramer staining and peptide-stimulation assays. Panel A shows the scatter plots of tetramer staining with five allele-specific tetramer reagents. Panel B represents data for peptide-stimulation assay that measures INF-*γ* production. Panel C corresponds to CD107a/b expression after stimulation with allele-specific peptides.

CMV-specific CD8^+^ T cells are typically present at very low frequencies in healthy CMV seropositive donors. In order to include a wider range of the tetramer positive cells in the analysis, PBMC were isolated from seropositive, HLA-matched donors, and the cell densities were adjusted to give 5-fold increase of tetramer positive cells compared to the number expected in the whole blood. For both HLA-tetramer staining and peptide assays, the samples were 2- or 3-fold diluted and then either directly stained with HLA-tetramers in a fixed volume and transferred into tubes with beads or stimulated with corresponding CMV-peptide in the presence of costimulatory antibodies and HLA-tetramers and analyzed for IFN-*γ* and CD107a/b production. Peptide stimulated samples were also transferred into tubes with beads in order to acquire a fixed number of the sample events. Both assays were carried out in triplicates.  Figure 1 shows the scatter plots with the results. All assays were found to be linear. 

### 3.4. Analytical Sensitivity

In order to determine the limit of detection of CMV-specific CD8^+^ T cells and their subsets producing IFN-*γ* and CD107a/b, we performed tetramer staining of whole blood and CMV-peptide stimulation of PBMC isolated from three CMV-seronegative donors. The average of positive cells was calculated for each assay, representing all five HLA alleles and corresponding CMV-epitopes (Table 4). Our reported sensitivity for tetramer staining is in range from 0.1 to 0.23 cells per uL of blood. The analytic sensitivity for IFN-*γ* and CD107a/b CMV-specific cells is in range from 0 to 0.23 and from 0.11 to 0.98 cells per *μ*L of blood correspondingly. 

### 3.5. Analytical Specificity

The analytical specificity was evaluated by comparing the numbers of CMV-specific tetramer positive cells and their subsets of IFN-*γ* and CD107a/b producing cells upon peptide stimulation, using samples from three HLA-mismatched and three HLA-matched CMV-seropositive donors. The absolute numbers of positive cells for representing each group of donors and HLA-alleles with corresponding CMV-epitopes are shown in Tables 5 and 6. No significant cross-reactivity was detected in the blood and peptide-stimulated samples from the allele mismatched donors. As expected, all the allele matched donors exhibited readily detectable, distinct subsets of CMV-specific CD8^+^ T cells producing IFN-*γ* and CD107a/b (Tables 5 and 6). The specificity level measured in samples from HLA-mismatched donors was lower than limit of detection. 

### 3.6. Reference Ranges

The study was carried out using whole blood samples obtained from 33 healthy CMV-seropositive individuals. The reportable ranges for HLA-tetramers staining derive from the direct staining of whole blood, while peptide stimulation data comes from stimulated PBMC as described in Materials and Methods. We measured CMV-specific CD8^+^ T cells using all 5 different tetramer reagents and CMV peptides restricted to HLA-A*01:01, A*02:01, B*07:02, B*08:01, and B*35:01 alleles. A total of 62 distinct HLA-restricted results (as some of the donors were positive for more than one tetramer) were collected for each of the assays. For HLA-tetramer staining the reference interval with 95% confidence level was 0 to 18 cells per *μ*L of whole blood. In terms of IFN-*γ* production, the reference interval was 0 to 2 cells per *μ*L of whole blood. The reference interval for CD107a/b CMV-specific CD8^+^ T cells was 0 to 3 cells per *μ*L of whole blood.

### 3.7. Monitoring CMV-Specific CD8^+^ T Cells and Determining Their Function and Cytolytic Activities in HSCT Patients

**Figure 2:**
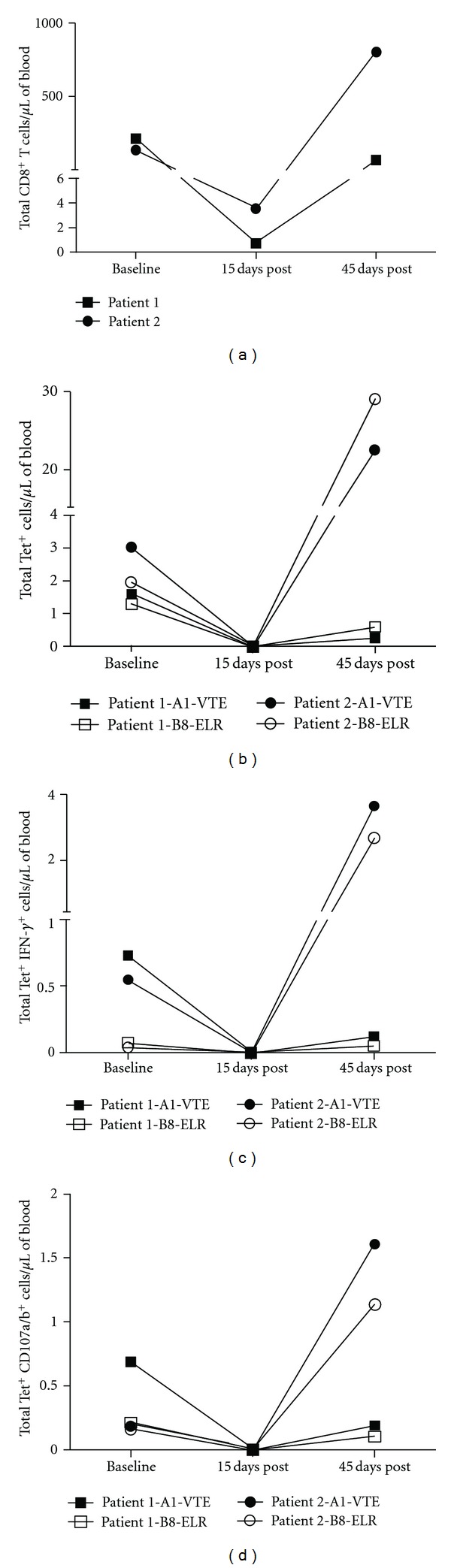
Results of CMV-related immune competence assays in two patients treated with autologous HSCT. Absolute counts of CD8^+^ T cells (a); CMV tetramer-specific CD8^+^ T cells (Tet^+^) (b); Tet^+^ IFN-*γ*-producing cells (c); Tet^+^ CD107a/b^+^ cells (d) are shown per microliter of blood. Results are presented for CMV-specific HLA-A*01:01 tetramer (A1-VTE), and HLA-B*08:01 tetramer (B8-ELR). Baseline counts correspond to results before transplantation.

We then examined CMV-specific CD8^+^ T cell immune recovery in two autologous HSCT recipients treated for multiple myeloma. Both patients were CMV seropositive prior to transplantation. Blood samples were collected before high-dose chemotherapy was administered for autologous HSCT, and then again on days 15 and 45 after stem cell infusion. The data is shown in Figure 2. Baseline counts represent each patient's CMV CD8^+^ T cell immune status before HSCT treatment. Both patients showed dramatic decrease in the number of CD8^+^ T cells and absence of CMV-specific CD8^+^ T cell immunity on day 15 post-HSCT. Recovery of CMV-specific CD8^+^ T cell numbers and their functional and cytolytic activities was evident at 45 days posttreatment in both individuals. However, the recovery of the cell populations was much more pronounced in one patient compared to the other one. In the latter, the absolute numbers of total CMV-specific CD8^+^ T cells and those producing IFN-*γ* and CD107a and CD107b remained below baseline values. 

## 4. Discussion

CMV is one of the most important opportunistic pathogens affecting HSCT and SOT recipients, with detrimental direct and indirect health effects [1–3]. Reactivation of the virus occurs in 70–80% seropositive patients, and if not treated, 20–35% of them will develop tissue-invasive CMV disease that is associated with significant morbidity and mortality (direct effects) [19]. The genome of CMV encodes a number of viral proteins that are able to downmodulate the host immune system and consequently facilitate the development of other opportunistic infections as well as allograft (indirect effects) [20]. Prevention of CMV disease posttransplant can be achieved with antiviral prophylaxis. Although proven to be effective, there are several concerns about antiprophylactic treatments. The drugs are only effective while the patient is taking them and the medication related side-effects are well documented [3]. Furthermore, CMV can also develop drug resistance after prolonged exposure to antiviral therapy.

Examining CMV-specific T cell immunity in patients who have undergone either HSCT or SOT is critical for a comprehensive medical assessment of patients who might be at risk of developing CMV disease. An ideal immune monitoring assay should provide measurements of T cell number and function. There are a variety of T cell assays for CMV immunity in experimental setting without clear clinical application [21]. Most assays rely on the detection of IFN-*γ* by ELISA or ELISPOTs after stimulation with CMV specific antigens. Test sensitivity in these assays decreases after transplantation due to lymphopenia and immunosuppression. Furthermore, these assays cannot differentiate CD4^+^ and CD8^+^ T cells. Approaches directed at evaluating CMV-specific T cell immune competence at the single-cell level, such as HLA-tetramer-based assays combined with intracellular cytokine staining upon peptide stimulation, overcome those issues. While enumeration of CMV-specific CD8^+^ cells with HLA-tetramers determines the absolute numbers of these cells in whole blood samples, intracellular cytokine staining, and staining of cytolytic markers provide an assessment of functionality of these cells. 

An ideal CMV-specific T cell immune competence assay for clinical use should be precise, reproducible, have a rapid turnaround time, and be robust enough to allow shipping of specimens to specialized referral laboratories. In this study, we focused our effort on validating three assays that are designed to measure the numbers of CMV-specific CD8^+^ T cells and determine their functional and cytolytic activities with respect to production of IFN-*γ* and CD107a/b molecules. The study was designed to determine the absolute number of cells in blood samples instead of simply measuring the cells frequencies. The rational for that is (1) total numbers of lymphocytes and all the other major subsets such as CD8^+^ T cells varies from one individual to another; (2) patients undergoing transplant treatment have dramatically reduced numbers of all lymphocytes subsets, which makes it difficult to ascertain the level of CMV immune competence of the recipient. We choose five HLA class I alleles (HLA-A*01:01, A*02:01, B*07:02, B*08:01, and B*35:01) that are found in >80% of the general population [22] as a starting point and plan to validated more alleles in the future. 

The analysis of data derived from the reproducibility studies of CMV-specific CD8^+^ T cell quantitations revealed a relatively wide range of CV values in both intra- and interassays (3 to 24%). Interestingly, comparison of CVs revealed an inverse correlation with the absolute counts of CMV-specific cells: the higher the cell count the lower CV was determined. Accordingly, higher CV values were found for CMV-specific CD8^+^ T cells expressing IFN-*γ* and CD107a and CD107b molecules (up to 60% for HLA-B*35:01), as they typically represented 10–50% of the total antigen-specific population. Similar observations were made in other studies [23]. Maecker and colleagues compared three assays (HLA-tetramer staining, intracellular cytokine staining, and ELISPOT) examining HLA-A*02:01 CMV responses restricted to a single epitope in three seropositive donors. Although they measured the cell frequencies instead of the absolute cell numbers, in their study, CVs of interassays was in range of 10–35% for tetramer staining and 7–50% for intracellular IFN-*γ* assay [23]. When the results from both studies are compared, one can conclude that the CV range in reproducibility studies is dependent on the size of a cell population rather than HLA allele restriction and associated epitope.

Linearity studies were challenging to carry out because of the small size of CMV-specific CD8 T cells that are normally present in the blood of healthy donors. To overcome this obstacle, we adjusted the cell density to obtain 5-fold increase of tetramer-specific cells per *μ*L of a sample. After serial dilutions of samples in both the tetramer and peptide stimulation assays, the frequencies of CMV-specific CD8 T cells and their subsets producing IFN-*γ* and CD107a/b compared to the total lymphocyte and CD8 T cell population remained the same (data not shown). This indicates that the reduction of the total number of lymphocytes and antigen-presenting cells after dilution has not affected the quality of the assays. The absolute cell counts that we have measured after the dilution were in different ranges with respect to HLA allele restriction and corresponding CMV-epitope. For the tetramer staining, the starting points were as high as 500 cells (HLA-A*01:01) and as low as 9 cells per ul of a sample (HLA-B*35:01). Linearity was found at all points measured. Similarly, the peptide stimulated samples had a very wide range, although somewhat lower than tetramer staining, and linear as well. Linearity over such a wide range makes both assays valid for clinical studies of healthy and infected individuals, as the later might exhibit much higher cell counts of CMV-specific CD8 T cells during periods of virus reactivation.

Analytical sensitivity is another important parameter in validation studies. It allows determining the minimum staining in intensity above nonspecific levels [24]. In the present study, we tested samples from 3 CMV-seronegative donors for each assay and the corresponding HLA-tetramers. Our data is in agreement with previously published studies of tetramer staining [25]. The limit of detection for tetramer staining and peptide stimulation assays were 0.10–0.23 (Tet^+^), 0–0.23 (IFN-*γ*), and 0.1–0.98 (CD107a/b) cells per ul of blood. These numbers have only scientific merit; the clinician using these assays ought to use integers in clinical studies for a clearer interpretation. 

The objective of analytical specificity studies is to determine the performance of a given reagent with its specific target [24]. In this study we used samples from HLA-mismatched, CMV-seropositive donors to determine the level of cross-reactivity for all three assays. Examples of correct specificity derived from experiments that were carried out with samples of HLA-matched, CMV-seropositive donors. Our results indicate very strong specificity levels for all assays and tetramer reagents as the absolute cell counts were lower than limits of detection. Rare examples of cross-reactive CD8 T cell epitopes and HLA alleles have been described in humans and mice [26, 27]. No such data were obtained in this study.

Determining the reference intervals provides important information regarding CMV-immune status of the patients. In this study, we investigated healthy CMV-seropositive donors, assuming that they would represent the numbers indicative of the level of protective CD8 T cell immunity to CMV reactivation. Reference intervals for tetramer staining (0–18 cells per *μ*L), IFN-*γ* (0–2 cells per *μ*L), and CD107a/b (0–3 cells per *μ*L) assays were determined with 95% confidence level, excluding the data from two donors. Those were defined as out-layers, showing 65.75 and 89.88 tetramer positive cells per *μ*L of blood. We think those unusually high numbers might indicate a recent asymptomatic CMV reactivation that has been documented previously [4]. Consistently lower numbers found for IFN-*γ* and CD107a/b CMV-specific CD8 T cells, compared to the tetramer positive cells, reflects the nature of latently persistent infections rather than advantage of one assay over another. Unresponsive, dysfunctional CD8 T cells specific to CMV and EBV latently persistent infections have been described extensively [18].

Finally, we examined the potential use of CMV-specific CD8^+^ T cell numbers and functional and cytolytic responses in two autologous HSCT recipients treated for multiple myeloma. Both patients were positive for CMV infection by serology at the time of transplantation. In both cases, no anti-CMV prophylaxis was given after transplant. Prevention of CMV disease was done with the early initiation of preemptive therapy based on the results of weekly plasma CMV quantitative real-time PCR testing. The results showed that both patients had a decrease in the number of CD8^+^ T cells below assay sensitivity and absence of CMV-specific CD8^+^ T cell functional and cytotoxic responses 15 days after autologous HSCT (Figure 2). This is expected due to conditioning therapy that patients undergo prior to transplant. Interestingly, testing done 45 days after transplantation revealed that one patient had a pronounced recovery of CMV-specific CD8^+^ T cell numbers and their functional and cytolytic activities, while in the other the absolute numbers of total CMV-specific CD8^+^ T cells and those producing IFN-*γ* and CD107a and CD107b remained below or close to baseline values (Figure 2). The difference in the speed of recovery of CMV-specific CD8^+^ T cell numbers and their functional and cytolytic activities between patients suggest that one patient might have recovered immunity against CMV infection much earlier that the other one. In both cases no evidence of CMV reactivation was documented by viral replication testing during the initial 45-day period posttransplant. Remarkably, the patient with slow recovery of CMV immunity developed CMV viremia at day 62 posttransplant that required preemptive therapy, whereas the patient with rapid recovery remains free of CMV reactivation up to one year posttransplant in the absence of antiviral prophylaxis. 

The results of CMV-specific CD8^+^ T cell immune competence and viral replication monitoring in these two patients suggest that combined use of these assays can guide antiviral therapy posttransplant. In this regard, a recent study using HLA tetramers for prediction of recurrent or persistent CMV infection or disease in allogeneic HSCT found that delayed recovery of CMV-specific T cells (<7 cells/mL during first 65 days after transplantation) is a significant factor for developing recurrent or persistent CMV infection compared to patients showing rapid recovery [25]. No functional or cytotoxic activity of CMV-specific CD8^+^ T cells was analyzed in those patients. More studies are needed to determine whether specific thresholds in number and functional activity of CMV-specific T cells can be used to guide prophylactic protocols. 

## 5. Conclusion

This study provides target values for CMV immune competence assays for the characterization of CD8^+^ T cell responses posttransplant measured in the absolute cell count per *μ*L of blood. The CV range in precision studies is dependent on the size of cell populations rather than HLA allele restriction and associated epitope. CMV tetramer-based functional and cytotoxic monitoring can be an important tool for clinicians to evaluate the risk of CMV disease posttransplant and guide prophylactic and preemptive therapeutic choices. 

## Figures and Tables

**Table 1:** Major HLA class I alleles and defined CMV HLA class I Epitopes.

HLA allele	CMV epitope	Name
HLA-A*01:01	VTEHDTLLY	A01-VTE
HLA-A*02:01	NLVPMVATV	A02-NLV
HLA-B*07:02	TPRVTGGGAM	B07-TPR
HLA-B*08:01	ELRRKMMYM	B08-ELR
HLA-B*35:01	IPSINVHHY	B35-IPS

**Table 2:** Precision within assays.

HLA allele and peptide	Tetramer staining		IFN-*γ*		CD107a/b	
	Average	CV%	Average	CV%	Average	CV%
A01-VTE	140.29	3.1	45.26	2.5	38.19	3.4
A02-NLV	9.44	5.6	2.52	15.8	2.84	15.6
B07-TPR	6.09	20	1.58	10	1.7	15.2
B08-ELR	5.79	15.2	0.84	34.9	1.31	8.2
B35-IPS	1.58	24	0.19	47	1.27	59.7

**Table 3:** Precision between assays.

HLA allele and peptide	Tetramer staining		IFN-*γ*		CD107a/b	
	Average	CV%	Average	CV%	Average	CV%
A01-VTE	152.61	7	48.45	6	40.36	5
A02-NLV	9.63	4	2.47	3	2.85	2
B07-TPR	6.19	10	1.45	15	1.44	16
B08-ELR	6.69	12	0.89	12	1.24	16
B35-IPS	2.69	5	0.19	4	1.52	48

**Table 4:** Analytical sensitivity. Values represent number of cells per *μ*L of blood.

HLA allele and peptide	Tetramer staining	IFN-*γ*	CD107a/b
A01-VTE	0.1	0.02	0.04
A02-NLV	0.19	0.23	0.98
B07-TPR	0.13	0.02	0.11
B08-ELR	0.23	0.11	0.34
B35-IPS	0.1	0	0.13

**Table 5:** Specificity of tetramer staining. Values represent number of cells per mL of blood.

Donors	A01-VTE	A02-NLV	B07-TPR	B08-ELR	B35-IPS
HLA-mistmatched donor 1	0.2	0.32	0.1	0.2	0.3
HLA-mistmatched donor 2	0.1	0.31	0.1	0.1	0.1
HLA-mistmatched donor 3	0.1	0.33	0	0.3	0.1

HLA-matched donor 1	65.75	5.67	2.4	6.05	1.41
HLA-matched donor 2	10.36	11.84	2.19	4.13	5.56
HLA-matched donor 3	7.08	7.68	17.24	5.77	3.57

**Table 6:** Specificity of peptide stimulation assays. Values represent number of cells per mL of blood.

Donors	A01-VTE		A02-NLV		B07-TPR		B08-ELR		B35-IPS	
	IFN-*γ*	CD107a/b	IFN-*γ*	CD107a/b	IFN-*γ*	CD107a/b	IFN-*γ*	CD107a/b	IFN-*γ*	CD107a/b
HLA-mistmatched donor 1	0	0	0	N/A	0.01	0.03	0.03	0.07	0.02	0.05
HLA-mistmatched donor 2	0	0.03	0.09	0.65	0.05	0.02	0.02	0.05	0	0.06
HLA-mistmatched donor 3	0	0.1	0.22	0.78	0.02	0.05	0.06	0.21	0	0

HLA-matched donor 1	2.33	1.92	1.17	3.96	0.33	0.48	0.62	1.77	0.39	1.57
HLA-matched donor 2	1.37	1.65	2.44	7.51	0.75	0.79	0.26	0.67	2.34	1.7
HLA-matched donor 3	0.71	0.73	1.08	4.84	3.32	1.86	0.33	0.46	0.32	1.04
